# New PET Radiotracers for the Imaging of Neuroendocrine Neoplasms

**DOI:** 10.1007/s11864-022-00967-z

**Published:** 2022-03-24

**Authors:** Emilia Fortunati, Giulia Argalia, Lucia Zanoni, Stefano Fanti, Valentina Ambrosini

**Affiliations:** 1 Nuclear Medicine, Department of Experimental, Diagnostic and Specialty Medicine, University of Bologna, Bologna, Italy; 2 Nuclear Medicine, IRCCS, Azienda Ospedaliero-Universitaria Di Bologna, Bologna, Italy

**Keywords:** Neuroendocrine tumours, PET/CT, Radiotracers, Theranostics

## Abstract

Neuroendocrine neoplasms (NEN) are a heterogeneous group of tumours derived from cells of neuroendocrine origin and can potentially arise everywhere in the human body. The diagnostic assessment of NEN can be performed using a variety of PET radiopharmaceuticals. Well-differentiated NEN (NET) present a high expression of SSTR (somatostatin receptors) and can therefore be studied with 68Ga-DOTA-peptides ([68Ga]Ga-DOTANOC, [68Ga]Ga-DOTATOC, [68Ga]Ga-DOTATATE). Current guidelines recommend the use of SSTR imaging to assess disease extension at staging/restaging, follow-up, assessment of response to therapy and selection of patients who may benefit from radionuclide therapy (PRRT). [18F]F-FDG is used for the assessment of high-grade tumours (high-grade G2, G3 and NEC) and in every case, there is one or more mismatched lesions between diagnostic CT (positive) and SSTR-PET/CT (negative). [18F]F-DOPA is currently used for the assessment of medullary thyroid carcinoma, neuroblastoma, primary pheochromocytoma and abdominal paraganglioma. In recent years, however, several new tracers were designed exploiting the many potential targets of the neuroendocrine cell and were employed in clinical trials for both imaging and therapy. Currently, the real-life clinical impact of these tracers is still mostly not known; however, the favourable biodistribution (e.g. [68Ga]Ga-FAPI, SSTR antagonists) and the possibility to use new theranostic pairs may provide novel diagnostic as well as therapeutic options (e.g. [68Ga]Ga-PSMA, [64Cu]Cu-SARTATE, [68Ga]Ga-CXCR4) for NEN patients.

## Introduction

NEN are a rare and heterogeneous group of tumours, for both primary tumour site and clinical behaviour over time. In fact, their clinical presentation varies from relatively indolent tumours (most frequently) to more aggressive forms showing rapid progression. The most common primary site is represented by the gastro-entero-pancreatic (GEP) tract (mostly pancreas and ileum) and the lungs [1•]. Most NEN are well differentiated (NET, neuroendocrine tumours) and express somatostatin receptors (SSTR) that represent the cellular target for currently approved radiopharmaceuticals used for both diagnosis and therapy. NET are further subdivided into three pathological grades: G1 (Ki-67 <2%), G2 (Ki-67 3-20%) and G3 (Ki-67 >20%), showing a more favourable behaviour as compared to poorly differentiated neuroendocrine carcinomas (NECs, small and large cells) [2•]. Lung tumours are still classified as typical and atypical forms [3].

Nuclear medicine plays a crucial role in the routine diagnostic and therapeutic (radionuclide therapy, PRRT) flow chart of NEN. On the diagnostic side, most NET are accurately studied with [68Ga]Ga-DOTA-peptides while the forms with variable-to-low SSTR expression can be detected by [18F]F-DOPA (neuroblastoma, medullary thyroid carcinoma, pheochromocytoma, abdominal paraganglioma) [4]. [18F]F-FDG is generally used in undifferentiated forms (NECs), in high-grade NET G2 and NET G3, to detect more aggressive clones that generally drive the patients’ prognosis. Moreover, [18F]F-FDG can be used to characterise lesions that do not show substantial [68Ga]Ga-DOTA-peptides uptake and, when mis-matched, represent a contraindication to PRRT. On the therapeutic side, [90Y]Y-DOTATOC and [177Lu]Lu-DOTATATE have been extensively used for target treatment of SSTR-positive NET and [177Lu]Lu-DOTATATE has been approved by European Medicines Agency (EMA) [5] and Food and Drug Administration (FDA) for routine treatment. PRRT efficacy was demonstrated in many different studies with limited toxicity and tremendous impact on the patients’ quality of life. Current guidelines recommend the use of [18F]F-DOPA [4] as first choice tracer for the assessment of medullary thyroid carcinoma, neuroblastoma, pheochromocytoma and abdominal paraganglioma.

The NET cell, however, offers several different targets that can be exploited to design novel radiopharmaceuticals for both imaging and therapy. In recent years, several new compounds were investigated in clinical trials with particular interest towards new potentially theranostic pairs that could be employed for both imaging and target therapy. The purpose of this review is to summarise the current indications of clinically employed radiopharmaceuticals (68Ga-DOTA-conjugated peptides and their theragnostic pairs) as well as to discuss the preliminary results of the use of new emerging (Table 1) for NEN.

**Table 1:** Emerging radiopharmaceuticals and their targets

Radiotracer	Targets	Advantages	Theranostic
**SSTR agonists**			
**[18F]F-AlF-NOTA-octreotide**	SSTR expression	- NET	No
		- High TBR	
**[18F]F-SiFAlin-TATE**	SSTR expression	- NET	No
		- Favourable tumour-to-liver and tumour-to-spleen ratios	
**[64Cu]Cu-SARTATE**	SSTR expression	- High tumour-to-liver ratio	Yes, [67Cu]Cu-SARTATE
		- Possibility of diagnostic studies and for prospective dosimetry for [67Cu]Cu-PRRT	
**SSTR antagonists (NODAGA-JR11; DOTA-JR11)**	SSTR expression	- Compared to analogues: recognise a higher number of SSTR-binding sites, lower dissociation rate, higher TBR, not internalised	Yes, [177Lu]Lu-DOTA-JR11
**Exendin**	GLP-1R expression	- Insulinoma	Yes, with the limit of high kidney uptake
		- High tumour-to-pancreas ratio	
**[18F]F-MFBG**	Norepinephrine transporter expression	- Neuroblastoma or paraganglioma/pheochromocytoma	No
		- High TBR	
**[68Ga]Ga-CXCR4**	C-X-C motif chemokine receptor 4 expression	- Dedifferentiated NEN	Yes
**[68Ga]Ga-PSMA**	PSMA expression	- Highly vascularised lesions	Yes
**[68Ga]Ga-FAPI**	Fibroblast activation protein expression	- Preliminary data show uptake in NEN	Yes
		- Favourable biodistribution	

Legend: *SSTR*, somatostatin receptor; *TBR*, tumour-to-background ratio; *GLP-1R*, glucagon-like peptide-1 receptor; *PSMA*, a transmembrane protein with enzymatic function as folate hydrolase-carboxypeptidase

## 68Ga-DOTA-conjugated peptides

### Diagnostic performance and theragnostic use

The first functional imaging diagnostic method to study NET was scintigraphy with radiolabelled SSTR analogues. Although this procedure represented a major breakthrough in the diagnostic flow-chart at first, it has been mostly replaced by novel beta-emitting radiopharmaceuticals targeting SSTR, when available. In fact, scintigraphy presents several limitations mostly due to a less favourable radiopharmaceutical biodistribution (especially at liver and bowel level) and lower spatial resolution (detection rate ranges between 50 and 100% [6]). The radiotracers for SSTR-PET/CT ([68Ga]Ga-DOTA-conjugated peptides) imaging are represented by [68Ga]Ga-DOTATOC, [68Ga]Ga-DOTATATE and [68Ga]Ga-DOTANOC [6] and represent the current gold-standard for imaging SSTR-expressing NEN. The superiority of SSTR PET/CT over both scintigraphy and conventional diagnostic procedure has been extensively reported, especially for the detection of small lesions, nodal and bone metastasis. A recently published umbrella review collected the results of 34 meta-analyses [7], analysing the use diagnostic performance of different radiopharmaceutical (SSTR PET/CT, DOPA PET/CT, FDG PET/CT) in NENs or suspected NET as well as the impact on clinical management. SSTR PET/CT showed a very high sensitivity and specificity in well-differentiated SSTR-expressing NET (>90%) and impacted clinical management in approximately 40% of cases. Although these radiopharmaceuticals present differences in the SSTR-binding affinity, they are considered clinically equivalent. Current guidelines [4, 8] recommend performing [68Ga]Ga-DOTA-conjugated peptides PET/CT for staging, re-staging after therapy and assessment of prognosis. Moreover, [68Ga]Ga-DOTA-conjugated peptides PET/CT is fundamental to demonstrate SSTR expression in vivo to select patients who might benefit from PRRT. Both DOTATOC and DOTATATE were used as theranostic compounds in clinical trials since the mid-nineties; however, [177Lu]Lu-DOTATATE was only recently approved. Moreover, although [177Lu]Lu-DOTATATE was originally approved for well-differentiated metastatic G1/G2 SSTR-positive mid-gut NET (NETTER-1 study) [9, 10], EMA/FDA extended its use also in pancreatic NET. The first multicentre, randomised phase 3 trial (NETTER-1) reported that progression-free survival (PFS) in the treated arm ([177Lu]Lu-DOTATATE PRRT + 30mg octreotide) was markedly longer than in the control-arm (FDA-approved off-label high-dose octreotide, 60mg) [9, 10]. PRRT should be considered a relatively safe treatment option. Among acute and short-term toxicity, nausea, vomiting and fatigue are common while patients report abdominal pain, diarrhoea and reversible mild haematologic toxicity less frequently. Alopecia and carcinoid crises are rare. Kidney and bone marrow are considered organs at risk for long-term PRRT toxicity: haematological reserve and renal function should be assessed before starting PRRT [11]. The phase III NETTER-1 study demonstrates that [177Lu]Lu-DOTATATE provides a substantial and clinically robust quality-of-life benefit for patients with progressive midgut NETs compared with high-dose octreotide [12••].

### Emerging issues

Although [177Lu]Lu-DOTATATE PRRT is currently used in routine clinical practice, many issues are still debated including patients’ selection criteria, the need of personalised treatment schemes, assessment of response to therapy, possibility of retreatment and when to use it in the therapeutic flow chart with respect to other treatment options. It is well known that PRRT achieves better results in patients presenting G1 and G2 tumours, due to their typically high SSTR expression. However, PRRT efficacy was also studied in patients with Ki-67>20%. Thang et al retrospectively evaluated 28 patients (Ki-67 ≤ 55%=22, Ki-67 > 55%=6; 17 had pancreatic, 5 small bowel, 3 large bowel, 2 bronchial and 1 unknown primary disease) treated with PRRT. Most patients showed FDG-positivity (25/28), received radiosensitising chemotherapy (20/28) and were treated for disease progression (89%). PRRT achieved a clinically relevant disease control with acceptable toxicity [13••], especially in patients with Ki-67 ≤ 55% in which PRRT might be considered as a first-line therapeutic option superior to platinum-based chemotherapy (provided the disease expresses high SSTR receptors with no discordant FDG-avid disease) [13••].

In a recent paper including 69 patients with NET G3 (primary sites: 46 pancreas, 11 unknown primary cancer, 6 midgut, 3 stomach, 3 rectum) treated with PRRT, PFS was significantly higher (11 vs 4 months) in patients with Ki-67 lower or equal to 55% (*n* = 53) as compared to G3 with higher Ki-67 levels. Moreover, 48 patients were also studied with baseline [18F]F-FDG-PET/CT: patients showing lesions with high [18F]F-FDG avidity (higher than the liver) presented shorter median PFS (7.1 months) and median OS (17.2 months) as compared to patients with lower [18F]F-FDG uptake (median PFS: 24.3 months and median OS: 41.6 months). In the setting of G3 cases, this study shows that [18F]F-FDG was useful to identify patients with high uptake on SSTR-PET/CT and absent or faint [18F]F-FDG avidity, which were associated with a better long-term prognosis [14••]. The on-going NETTER-2 clinical trial aims in fact to assess the efficacy of [177Lu]Lu-DOTATATE PRRT as first-line treatment in patients with G2 and G3 GEP-NET compared to treatment with high dose (60 mg) long-acting octreotide [15••].

Another issue of debate regards [18F]FDG-PET/CT: it is currently not part of the standard pre-PRRT administration protocol, and the combined use of SSTR-PET/CT and [18F]FDG-PET/CT was proposed to better select patients who might benefit more from target therapy and to exclude from treatment those who present spatially mis-matched [18F]F-FDG/SSTR lesions. In view of the clinically relevant data provided by [18F]FDG-PET/CT, Chan et al developed the “NETPET score” that stratifies patients on the basis of the relative SSTR/[18F]F-FDG uptake: this score was proved to be prognostic and helpful to select PRRT candidates [16•].

Treatment scheme personalisation is another area of debate. The NETTER-1 trial promoted the standard use of a fixed-dose scheme for [177Lu]Lu-DOTATATE PRRT administration (7.4GBq x 4) in clinical practice. However, Sansovini et al reported (in 63 patients with histologically confirmed unresectable or metastatic G1-G2 pancreatic NET) that the use of customised activity (especially in selected patients at risk of developing serious adverse sequelae) had limited side effects and resulted in good tumour control [17]. Moreover, significantly longer PFS and OS were observed in patients in whom the 23 Gy dose limit to the kidney was reached [18]. Moreover, only limited data are currently available regarding PRRT retreatment (consisting in the administration of additional PRRT-courses in relapsing patients after response to the first PRRT course). In a cohort of 133 patients with gastrointestinal (GI, 62%), pancreatic (23%) and bronchopulmonary (BP, 11%) NEN treated with the first course of PRRT, PFS was respectively 30, 19 and 12 months. The G1 and G2 tumours had PFS of 25 and 22 months, compared to 11 months in G3-NEN. A minority of patients were offered subsequent PRRT treatment (36 patients had a second PRRT course while 8 patients received even a third course). After a second PRRT course, patients with G1 and G2 tumours showed a PFS of 19 and 22 months, respectively. The G3 tumours (only three patients re-treated) had a PFS of 4 months. This study demonstrated a favourable response also after a second course of PRRT, with a progression-free survival of 19 months across all primary tumours. The decision to retreat patients with a 2nd or even a 3rd course was mainly based on combination of the duration of response, toxicity and degree of uptake on SSTR-PET/CT. Patients who developed progression within 12 months were excluded from retreatment [19••]. In a recent meta-analysis, 13 studies described that PRRT retreatment was associated with an acceptable safety profile and promising efficacy: in patients with progressive GEP-NET who received retreatment with [177Lu]Lu-DOTATATE, median PFS was ≥ 12 months from the time of re-treatment [20].

In order to improve PRRT efficacy, the administration of the tracer directly into the hepatic artery [21] was suggested. In particular, patients with hepatic dominant metastases would benefit from this approach due to an increase of uptake of the radiopharmaceutical [22]. Furthermore, [177Lu]Lu-DOTATATE PRRT was also proposed in the neoadjuvant setting to reduce tumour size, eventually rendering patients candidates for surgery [23•]. In the rare setting of paragangliomas, preliminary results demonstrate the superiority of [177Lu]Lu-DOTATATE PRRT compared to [131I]I-MIBG treatment [24]; however, the use in this clinical setting is still investigational.

## [18F]F-AlF-NOTA-octreotide

Although 68Ga labelling offers the advantage of eluting the isotopes from a commercially available generator, therefore bypassing the need of an on-site cyclotron, on the other hand, the possibility to use fluorine labelling offers the advantage of a longer half-life, a better spatial resolution and a higher production yield with consistent benefits in imaging and logistical field. Recently, a novel SSTR agonist labelled with fluorine was synthesised: [18F]F-AlF-NOTA-octreotide, produced with the Al18F-method, with the advantages of a chelator-based radiolabelling method and the advantages of fluorine-18 in imaging and logistical field [25]. Preliminary data in 22 patients with proven NEN (16 GEP NEN: 2G1, 7G2, 7G3; 2 paragangliomas, 1 lung, 1 head/neck, 2 unknown primary carcinoma) compared [18F]F-AlF-NOTA-octreotide-PET/CT with [18F]F-FDG-PET/CT performed within 2 weeks. After 90 min, the radiotracer’s localisation augmented in kidneys and bladder, while the spleen demonstrated the highest uptake. Physiological uptake was also detected at pituitary, thyroid, adrenal glands, uncinate process of the pancreas, stomach and intestine level. Brain, lung, muscle and bone showed a low background activity and spleen got the highest dose, as the urinary bladder. Images showed optimal contrast, with a significantly high tumour-to-background ratio. The brain uptake of [18F]F-AlF-NOTA-octreotide was low thanks to the intact blood-brain barrier, an important aspect for brain metastasis detection. The [18F]F-AlF-NOTA-octreotide tumour uptake was significantly higher than [18F]F-FDG uptake, depending on the differentiation grade: [18F]F-AlF-NOTA-octreotide SUVmax was higher in well-differentiated NET than in poorly differentiated tumours. Few tumours showed a “flip-flop” phenomenon, with variable affinity for both radiotracers at different sites: higher Ki-67 values were observed in [18F]F-FDG-avid lesions, while lower values were detected in [18F]F-AlF-NOTA-octreotide avid tumours [26••]. In a limited sample of six healthy volunteers and six NEN patients, [18F]F-AlF-NOTA-octreotide-PET/CT was compared to [68Ga]Ga-DOTA-TATE-PET/CT performed within 6 months. The physiological uptake pattern was similar for both tracers, with lower uptake in most organs and bones for [18F]F-AlF-NOTA-octreotide and higher uptake in salivary glands for [68Ga]Ga-DOTATATE. [18F]F-AlF-NOTA-octreotide tumour uptake was lower in tumour lesions but increasing over the time, as demonstrated by all lesions’ mean SUVmax progressive increase. Another important finding is the lower liver background uptake for [18F]F-AlF-NOTA-octreotide, which allowed it to identify more liver lesions as compared to [68Ga]Ga-DOTA-TATE. On the contrary, notwithstading the slightly lower background activity in the bone, [18F]F-AlF-NOTA-octreotide missed more bone lesion than [68Ga]Ga-DOTA-TATE, in particular in patients with a high number of bone lesions. Overall, these preliminary data (favourable biodistribution, dosimetry and tumours targeting) indicate [18F]F-AlF-NOTA-octreotide as a promising tracer for NET imaging [27••]. A further retrospective analysis, including 128 patients with proven or suspected NEN (27 G1, 48 G2, 43 G3/NEC), also confirmed the favourable biodistribution, higher uptake in well differentiated tumours (G1/G2 vs G3) and in tumour lesions as compared to benign lesions (i.e. fractures or inflammatory lesions). Some G3 showed [18F]F-AlF-NOTA-octreotide uptake and no uptake with [18F]F-FDG, highlighting the importance of double tracer imaging [28•].

## [18F]F-SiFAlin-TATE

[18F]F-SiFAlin-TATE is a novel and promising somatostatin analogue radiotracer labelled with 18F, showing the abovementioned advantages of fluorine-labelling (longer half-life, a better spatial resolution and a higher production yield with consistent benefits in imaging and logistical field) and the availability of a kit for labelling. The first-in-human [18F]F-SiFAlin-TATE-PET/CT scan was reported in 2019 in a patient with metastatic NET with unknown primary, presenting liver metastasis with Ki-67 of 5% and treated with emi-hepatectomy. Previous [68Ga]Ga-DOTATATE-PET/CT showed uptake in cardiac and bone metastasis, and PRRT was planned. [18F]F-SiFAlin-TATE-PET/CT performed prior to PRRT was comparable to [68Ga]Ga-DOTATATE-PET/CT for cardiac and bone metastasis, as well as the quantitative SPECT/CT performed after the first PRRT cycle, supporting its future use as an alternative to [68Ga]Ga-DOTA-conjugate-peptides-PET/CT [29••]. In a retrospective study including 13 patients with NEN (ileum=5, pancreas=2, lung=2, duodenal=1, colon=1, stomach=1, unknown=1; G1: 7 and G2: 6) studied with both [68Ga]Ga-DOTA-TOC and [18F]F-SiFAlin-TATE-PET/CT, tracers’ biodistribution, tumour uptake and image quality were studied. [18F]F-SiFAlin-TATE biodistribution was higher, though not significantly, in the liver, adrenal glands and spleen. Significantly higher uptake was described, instead, in kidneys. Tumour uptake was calculated by SUVmax and SUVmean measurement, both in [18F]F-SiFAlin-TATE-PET/CT and [68Ga]Ga-DOTA-TOC-PET/CT, in 109 lesions (32 were liver metastasis, 28 bone, 24 lymph node, 7 subcutaneous, 7 peritoneal, 4 pleural, 2 lung, 3 myocardial and 2 ovarian metastasis): with the exception of lung lesions, a significantly higher [18F]F-SiFAlin-TATE tumour uptake was described in almost all tumour lesions. In comparison to [68Ga]Ga-DOTATOC, the higher tumour uptake led to a favourable tumour-to-liver and tumour-to-spleen ratios. Despite the heterogeneous population including different primary lesions undergoing various treatments, these preliminary data support the potential utility of [18F]F-SiFAlin-TATE [30••].

## [64Cu]Cu-SARTATE

64Cu is a longer life positron-emitting radioisotope with a half-life of 12.7h. It is important to evaluate a predictive dosimetry and patients adequacy for [67Cu]Cu-PRRT, which is a β-emitting radionuclide with positive qualities for its use in therapy. Further good qualities of 64Cu are the favourable positron energy and production by cyclotron. It is reported a first-time-in-humans trial of [64Cu]Cu-MeCOSar-Tyr3-octreotate ([64Cu]Cu-SARTATE) which enrolled 10 patients with proven G1 or G2 NEN and a previous documented positivity on [68Ga]Ga-DOTATATE-PET/CT. Images were acquired at 30 min, 1h, 4h and 24h after [64Cu]Cu-SARTATE administration. The aim of this trial was to evaluate the high late-retention in tumour and clearance from the liver, to suggest suitability for prospective dosimetry for [67Cu]Cu-SARTATE PRRT. In most patients, image quality at 1h after injection of [64Cu]Cu-SARTATE can be considered comparable to that of [68Ga]Ga-DOTATATE at the same time. It is worth noticing how the lesion-to-liver ratio increased progressively in [64Cu]Cu-SARTATE scans between 4 and 24 h. So, delayed imaging may allow better lesion detection and improve sensitivity at liver level (a frequent metastasis site of NEN). All these aspects make [64Cu]Cu-SARTATE a safe PET/CT radiotracer, with good qualities for diagnostic studies and for prospective dosimetry for [67Cu]Cu-PRRT [31••]. Considering the few clinical reports, it is also interesting to evaluate the preliminary results portrayed in preclinical results. Cullinane et al. described, in the exocrine pancreatic tumour setting, that [67Cu]Cu-SARTATE has similar efficacy compared to [177Lu]Lu-TATE, with a significantly shorter half life which produces a higher dose-rate and with an elevated effectiveness [32]. Dearling et al. evaluated the possibility to detect and treat minimal residual disease of neuroblastoma in nude mice, showing the potential use of the theranostic pair of [64/67Cu]Cu-SARTATE [33].

## SSTR antagonists

SSTR antagonists rather than agonists have the potential to improve SSTR-PET/CT imaging because they recognise a higher number of SSTR-binding sites, are not internalised and have a lower dissociation rate. In a prospective phase I study, [68Ga]Ga-OPS202 (NODAGA-JR11), a SSTR2 antagonist for PET/CT imaging, was investigated. It presented a high receptor’s affinity, an acceptable radiation dose to organs and a low background activity (especially in the liver and gastrointestinal tract) [34]. In the subsequent prospective phase II study, they compared [68Ga]Ga-OPS202 and [68Ga]Ga-DOTATOC in patients with GEP NET. The antagonist showed substantially higher TBRs (tumour-to-background) and sensitivity at liver level. For malignant lymph nodes, there were no substantial differences between the two radiotracers in the TBR, regardless of the reference tissue [35•]. Regarding bone metastasis, the antagonist showed lower detection rate. Interestingly, [68Ga]Ga-DOTA-JR11 showed lower uptake than [68Ga]Ga-DOTATATE in normal organs such as spleen, renal cortex, adrenal glands, pituitary glands, stomach wall, normal liver parenchyma, small intestine, pancreas and bone marrow [36]. The corresponding therapeutic radiotracer, [177Lu]Lu-OPS201 (DOTA-JR11), demonstrated higher tumour radiation doses per administered activity and similar or lower radiation doses to normal organs as compared to [177Lu]Lu-DOTATATE [37]. Yordanova et al. confirm the largely concordant uptake from [68Ga]Ga-DOTA-JR11-PET and post-[177Lu]Lu-DOTA-JR11-SPECT/CT, making [68Ga]Ga-DOTA-JR11 and [177Lu]Lu-DOTA-JR11 a suitable theranostic pair [38•]. [177Lu]Lu-DOTA-LM3 is another recently reported radiopharmaceutical used for treatment: the first-in-human study in patients with metastatic NET reported an excellent tumour response with a disease control rate of 85.1% (attributable to the high doses delivered to the metastases) and a low nephrotoxicity and haematotoxicity [39••].

Theoretically, the major advantage of the use of the antagonists could lie in the possibility to improve detection of tumours presenting with a lower SSTR expression and to improve the detection of metastasis at liver level, thanks to the lower background.

## Exendin

Insulinoma is one of the most common types of functional pancreatic NET arising from pancreatic β-cells. Clinically, it is characterised by hyperinsulinemia associated with hypoglycaemia. Since surgery is the main-stay of the treatment of insulinoma, the localisation of the neoplastic mass is of primary importance. However, the detection of the tumour is often challenging because insulinomas are often small sized and multifocal, and conventional imaging (CT, MRI) often show low sensitivity [40]. Moreover, only a minority of insulinomas express SSTR and can be detected by DOTA-peptides tracers. On the contrary, emerging evidence supports the use of radiotracers binding Glucagon-like peptide-1 receptor (GLP-1R), a G-protein-coupled receptor expressed on pancreatic beta cells and overexpressed in insulinoma. The instability of native GLP-1 (because of its rapid degradation by the enzyme dipeptidyl peptidase IV) makes it unsuitable for radiolabeling; therefore, Exendin-4, an agonist with strong binding affinity for GLP-1R, was synthesised. Gallium-68 was used for labelling NOTA-MAL-Cys39-exendin-4 [40]: [68Ga]Ga-NOTA-MAL-Cys39-exendin-4 demonstrated high accuracy for insulinoma localisation, with a high tumour-to-pancreas ratio. Literature data indicated that [68Ga]Ga-NOTA-MAL-Cys39-exendin-4 detection rate may decrease in case of small lesions, in case of malignant insulinomas (in which SSTRs are expressed with high density, whereas GLP-1 receptor expression is decreased or absent) and for lesions in the distal pancreas tail, next to the left kidney (site of physiological radiotracer excretion) [41••]. Exendin-4-based tracers for therapeutic applications have also been developed but, due to high kidney uptake, renal toxicity is a serious concern that currently limits its clinical application [42]. Preliminary preclinical evidence suggests that renal toxicity may be reduced, employing a novel exendin-4-based PET radiotracer conjugated with polyethylene glycol (PEG) ([18F]FB(ePEG12)12-exendin-4), that showed high tumour uptake and rapid kidney clearance [43•].

## [18F]F-MFBG

[18F]F-meta-fluorobenzylguanidine ([18F]F-MFBG) is a fluorinated analogue of [123I]I-MIBG, labelled with 18F (half life of 110 min), which is accumulated in cells through the same norepinephrine transporter uptake mechanism of [123I]I-MIBG. The latter is currently clinically employed for NEN imaging and its major clinical applications are represented by imaging neuroblastoma, for both staging and follow-up. In the diagnostic setting, [123I]I-MIBG has been largely replaced by [18F]F-DOPA, also considering that approximately 10% of neuroblastoma are [123I]I-MIBG-negative. [123I]I-MIBG imaging however has a crucial role for the selection of patients candidate to nuclear therapy with [131I]I-MIBG. Although clinically useful, [123I]I-MIBG presents several limitations, including a poor imaging resolution and low quantitative accuracy [44••, 45]. The scan acquisition is long (particularly unfavourable considering that most patients are children), because images are acquired 20–24h after injection of [123I]I-MIBG. Patients’ preparation is complicated since many drugs interfere with uptake and retention of [123I]I-MIBG, as tricyclic antidepressants, sympathomimetics and antihypertensives (children can be hypertensive) and should be therefore withdrawn. Moreover, thyroid blockage is compulsory, with the aim of preventing thyroid uptake of free radioactive iodide from the [123I]I-MIBG [46]. To overcome such issues, [18F]F-MFBG could be a successful alternative. The first-in-human prospective study of [18F]F-MFBG-PET/CT imaging involved ten patients with proven neuroblastoma or paraganglioma/pheochromocytoma [44••]. Preliminary data showed that [18F]F-MFBG, biodistribution included substantial activity in the blood pool, liver, salivary glands (that decrease with time) and a principal excretion by urinary tract (clearance resulted faster than [123I]I-MIBG). [18F]F-MFBG was overall well tolerated, with a distribution similar to [123I]I-MIBG. [18F]F-MFBG-PET/CT demonstrated high tumour-to-background ratios with optimal detection of lesions in bones and in soft-tissues (without substantial uptake difference between 1–2h and 3–4h after injection). Despite modest uptake in the liver, lesions were detectable with relatively high contrast at 3–4h after [18F]F-MFBG injections. Five additional lesions were detected at 3–4h post-injections as compared to 1–2-h imaging at liver level. Imaging at 1–2h after injection demonstrated the highest tumour-to-background ratios (TBR) for bone lesions and soft-tissue and this would be the optimal time point for imaging, providing the better high-contrast images and good lesion detection [44••]. In a report of a single case in a patient with metastatic pheochromocytoma, [18F]F-MFBG-PET/CT imaging was acquired 60min after radiotracer injection and a clinical [123I]I-MIBG scan was performed 27h post-injection. [18F]F-MFBG-PET/CT confirmed an optimal tumour targeting with better image quality than [123I]I-MIBG scans. This patient had already performed a [68Ga]Ga-DOTATATE-PET/CT, as a part of work up before the radionuclide therapy. The lower uptake on adrenal glands shown by the [18F]F-MFBG-PET/CT, compared to [68Ga]Ga-DOTATATE-PET/CT, allowed the identification of primary lesions located in adrenal glands [47•].

## [68Ga]Ga-CXCR4

According to the literature C-X-C motif chemokine receptor 4 (CXCR4) is overexpressed in more aggressive and dedifferentiated NEN. Usually in the setting of tumours that poorly express SSTR, [18F]F-FDG is the PET tracer of choice. Weich et al. described the usefulness of CXCR4-directed imaging with the novel PET tracer [68Ga]Ga-Pentixafor as an alternative to [18F]FDG in poorly differentiated NEC; 11 patients were enrolled (the primary tumour, when identified, was located in the stomach, pancreas, oesophagus, ileum and rectum). [68Ga]Ga-Pentixafor positivity is associated with adverse prognosis, early progression and shortened overall survival [48••]. In fact, CXCR4 can be considered as a “tumour driver” which regulates the microenvironment and the dissemination of metastasis. In fact, [68Ga]Ga-Pentixafor spleen uptake in solid tumour patients correlates with the suppression of antitumour immune responses. In the setting of NEN, high spleen uptake is correlated with an elevated leukocyte count and thrombocytosis possibly attributed to high tumour metabolism [49]. CXCR4 can also be labelled with the cytotoxic beta-emitters such 177Lu or 90Y generating the novel theranostic agent Pentixather for CXCR4-directed endoradiotherapy [50•], which has already been used, with varying degrees of success, in multiple myeloma and other haemato-oncological diseases [51]. Since this therapy leads to bone marrow ablation, further studies are needed to evaluate its utility in NET.

## [68Ga]Ga-PSMA

Immunohistochemistry shows that prostate-specific membrane antigen (PSMA), notwithstanding its name, can be also overexpressed on the endothelial cells of the neo-vasculature of several types of solid tumours as well-differentiated thyroid carcinoma, lung adenocarcinoma, gastric/colon adenocarcinoma and renal carcinoma. Consequently, since [68Ga]Ga-PSMA first clinical use in prostate cancer patients, there is emerging evidence of its potential capacity to accidentally identify non-prostatic tumours [52]. Few reports indicate that [68Ga]Ga-PSMA can detect NET, typically characterised by [53•]. Two clinical cases of high [68Ga]Ga-PSMA focal uptake in the pancreas in patients with previous prostate cancer are known, which were later confirmed to be synchronous NET. Therefore, when evaluating a patient with prostate cancer, the incidental detection of PSMA-avid lesions of synchronous NET is possible [53•, 54•]. PSMA expression was also reported in two cases of medullary thyroid carcinoma (MTC) [55, 56], a condition generally challenging to treat: the use of [177Lu]Lu-DOTATATE-PRRT is only feasible in MTC presenting high SSTR (which is not common). Therefore, a potential, still unexplored treatment option could be represented by [177Lu]Lu-PSMA-PRRT in MTC cases showing substantial [68Ga]Ga-PSMA uptake [57•]. In further two cases of paragangliomas, which are usually asymptomatic and rare, [68Ga]Ga-PSMA uptake was also reported: these preliminary observations could offer a new therapeutic option for inoperable paragangliomas [58, 59]. Finally, another case report of prostate adenocarcinoma described [68Ga]Ga-PSMA uptake in a lesion with a clinical suspicion of pheochromocytoma showing also intense uptake in [68Ga]Ga-DOTA-peptide-PET/CT [60]. These preliminary data on [68Ga]Ga-PSMA indicate that it is important to consider the possibility of [68Ga]Ga-PSMA uptake in tumour entities beyond prostate cancer, even NEN, especially in patients showing [68Ga]Ga-PSMA uptake at sites that are uncommon for prostate cancer spread [61].

## [68Ga]Ga-FAPI

[68Ga]Ga-FAPI is an emerging and promising radiotracer for PET/CT imaging, showing excellent preliminary results in various tumour entities [62]. FAPI is a fibroblast activation protein (FAP) inhibitor overexpressed by fibroblasts and associated with cancer and poor prognosis. Different kinds of tumours express strongly FAP, especially epithelial carcinomas. Radiopharmaceuticals with FAP-targeting were recently studied and are radiolabelled FAP-specific inhibitors (FAPI). In particular, biodistribution of [68Ga]Ga-FAPI is very low as compared to other tracers, including [18F]F-FDG, improving the detection of lesions at brain, liver, head and neck, peritoneum, mesentery, omentum and axial skeleton level. Among all the FAPI tracers, FAPI-04 showed improved FAP binding and pharmacokinetics [63–65]. A single-centre retrospective analysis [66••] studied [68Ga]Ga-FAPI-PET/CT in 55 patients with rare tumours: cancer of unknown primary (*n* = 10), head and neck cancer (*n* = 13), gastrointestinal and biliary-pancreatic cancer (*n* = 17), urinary tract cancer (*n* = 4), neuroendocrine cancer (*n* = 4, although grade and primary site were not specified) and others (*n* = 7). Scans were acquired 60 min after tracer administration and three different [68Ga]Ga-labelled FAP ligands were used: FAPI-04, FAPI-46 and FAPI-74. At semiquantitative evaluation, a quite high value of SUVmax was measured in NET lesions, compared to others. One of the four patients enrolled by Dendl et al presented neuroendocrine metastasised urothelial bladder tumour showing high uptake both at 10 min after [68Ga]Ga-FAPI injection and after 1 h. This demonstrated the ability of [68Ga]Ga-FAPI-PET/CT to provide early diagnostic images [66••]. Kratochwil et al enrolled 3 NET patients in a retrospective study and described, despite a weak desmoblastic reaction observed at the histological examination, an intermediate [68Ga]Ga-FAPI uptake [62]. Several case reports indicated substantial uptake of [68Ga]Ga-FAPI in NET of different origins. A case of G2 (Ki-67: 15–20%) NET of the pancreas with hepatic metastasis underwent [68Ga]Ga-FAPI-04-PET/CT after [18F]F-FDG-PET/CT. [68Ga]Ga-FAPI-04-PET/CT showed high uptake both at pancreas level and as well as in a [18F]FDG-negative liver metastasis (thanks to the usually low liver background FAPI uptake) [67•]. Another G2 pancreatic NET (Ki-67: 10%) was studied with three radiopharmaceuticals: [68Ga]Ga-FAPI-04-PET/CT, [18F]F-FDG-PET/CT and [68Ga]Ga-DOTATATE-PET/CT. Tumour to liver ratio in [68Ga]Ga-FAPI-04-PET/CT was higher compared to the other tracers although [68Ga]Ga-DOTATATE could better detect a higher number of small liver metastatic lesions [68•]. Finally, Ergül et al described that [68Ga]Ga-FAPI-04-PET/CT uptake could be higher than [18F]F-FDG-PET/CT in liver NEC (Ki-67: 80%), suggesting a potential use for [68Ga]Ga-FAPI-04-PET/CT in NEC cases with low uptake on [18F]F-FDG-PET/CT [69•]. In conclusion, although very preliminary, these data indicate that FAPI is a tracer with favourable biodistribution in NEN: further studies are needed to better ascertain whether it could provide additional value in the clinical management of NEN (including both diagnosis and therapy) with elevated content of activated fibroblasts [70].

## Conclusions

In clinical practice, agonists of the SSTR are employed for both diagnosis and therapy (PRRT) of well-differentiated NEN. In high grade G2, G3 and NEC, [18F]F-FDG is currently employed. [18F]F-DOPA is preferred in particular types of NEN such as medullary thyroid cancer, pheochromocytoma and abdominal paraganglioma.

In recent years, emerging evidence supports the potentially promising role of new radiopharmaceuticals that exploit different targets on the NET cells. Preliminary reports are mostly encouraging, indicating a more favourable biodistribution (e.g. [68Ga]Ga-FAPI, SSTR-antagonists), accuracy in particular settings (e.g. Exendin superiority over SSTR-agonist in the setting of insulinoma; [18F]F-MFBG superiority over [123I]I-MIBG) or potential theranostic use (e.g. [68Ga]Ga-PSMA, [64Cu]Cu-SARTATE, [68Ga]Ga-CXCR4). However, the real-life clinical impact of these new radiopharmaceuticals is still mostly not known as a consequence of the limited number and very heterogenous cohorts of studied patients (in terms of primary site, Ki-67 levels, on-going treatments juts to name a few). Further studies are needed to better ascertain the clinical settings in which these tracers will provide an added value and definitive clinical impact.
